# Histopathological Image Diagnosis for Breast Cancer Diagnosis Based on Deep Mutual Learning

**DOI:** 10.3390/diagnostics14010095

**Published:** 2023-12-31

**Authors:** Amandeep Kaur, Chetna Kaushal, Jasjeet Kaur Sandhu, Robertas Damaševičius, Neetika Thakur

**Affiliations:** 1 Chitkara University Institute of Engineering and Technology, Chitkara University, Rajpura 140401, India; 2 Department of Applied Informatics, Vytautas Magnus University, 53361 Akademija, Lithuania; 3 Junior Laboratory Technician, Postgraduate Institute of Medical Education and Research, Chandigarh 160012, India

**Keywords:** breast cancer diagnosis, deep mutual learning, histopathology imaging diagnosis

## Abstract

Every year, millions of women across the globe are diagnosed with breast cancer (BC), an illness that is both common and potentially fatal. To provide effective therapy and enhance patient outcomes, it is essential to make an accurate diagnosis as soon as possible. In recent years, deep-learning (DL) approaches have shown great effectiveness in a variety of medical imaging applications, including the processing of histopathological images. Using DL techniques, the objective of this study is to recover the detection of BC by merging qualitative and quantitative data. Using deep mutual learning (DML), the emphasis of this research was on BC. In addition, a wide variety of breast cancer imaging modalities were investigated to assess the distinction between aggressive and benign BC. Based on this, deep convolutional neural networks (DCNNs) have been established to assess histopathological images of BC. In terms of the Break His-200×, BACH, and PUIH datasets, the results of the trials indicate that the level of accuracy achieved by the DML model is 98.97%, 96.78, and 96.34, respectively. This indicates that the DML model outperforms and has the greatest value among the other methodologies. To be more specific, it improves the results of localization without compromising the performance of the classification, which is an indication of its increased utility. We intend to proceed with the development of the diagnostic model to make it more applicable to clinical settings.

## 1. Introduction

**Figure 1:**
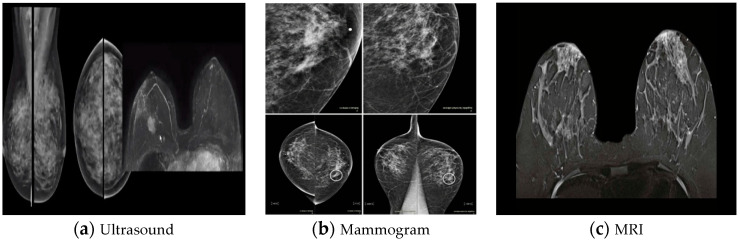
Medical imaging modalities for breast tissue: (**a**) ultrasound, (**b**) mammogram, and (**c**) MRI [10].

Breast cancer (BC) is the most common kind of cancer in women, accounting for around 30% of all new cancer diagnoses; it is also the second most fatal malignancy after lung and bronchial cancers [1]. According to the most recent data from the International Agency for Research on Cancer, which is part of the World Health Organization, breast cancer has exceeded lung cancer as the most frequent cancer, with 2.26 million new cases in 2020, overtaking lung cancer. It presents a major threat to the lives and health of women. Early diagnosis is crucial in the fight against cancer, and this can only be achieved with a reliable detection system. Two techniques that have been developed to aid in the diagnosis of breast cancer are medical image processing and digital pathology, respectively [2,3,4]. BC has two particularly alarming features among the many forms of cancer: being the most frequent disease in women across the globe and having a much higher fatality rate than additional kinds of cancer, because the histopathological examination is the most often utilized approach for the diagnosis of breast cancer. In many cases, pathologists still use the visual evaluation of histological samples beneath the microscope to make a diagnosis. Automated histopathological image classification is a study area that might speed up and reduce the risk of mistakes in BC diagnosis [5]. Histopathology uses a biopsy to obtain images of the diseased tissue [6,7]. Early identification is significant for illness treatment and a safer prognosis [8]. Noninvasive BC screening procedures include clinical breast assessment and tomography tests, such as magnetic resonance, ultrasound, and mammography. However, the requirement for verifying the identification of BC is the pathological study of a slice of the suspicious region by a diagnostician. Glass slides tarnished with hematoxylin and eosin are used to examine the microscopic details of the questionable tissue [9]. There are various analytical modes used for BC detection. Some of the general modes are mammography, magnetic resonance imaging (MRI), positron emission tomography (PET), breast ultrasound, surgery, or fine-needle aspiration to target the nerve of the alleged area (histopathological images), etc., as shown in Figure 1 [10,11].

Different methods, such as rule-based and machine-learning approaches, are used to evaluate breast cancer digital pathology images [12]. Recently, it has been shown that deep-learning-based approaches, which automate the whole processing, outperform classical machine-learning techniques in numerous image-assessment tasks [13]. Successful applications of convolutional neural networks (CNNs) in medical imaging have allowed for the early diagnosis of diabetic retinopathy, the prediction of bone disease and age, and other problems. Earlier deep-learning-based functions in histological microscopic image processing have demonstrated their capacity to be effective in the detection of breast cancer. Machine learning has played an increasingly important role in breast cancer detection over the last several decades. Several probabilistic, statistical, and optimization strategies could be used in the machine-learning approach to derive a classification model from a dataset [14].

Breast carcinoma is a commonly classified histopathology established on the selection of morphological aspects of the cancers, with 20 main cancer categories and 18 lesser subtypes. Invasive ductal carcinoma (IDC) and invasive lobular carcinoma (ILC) are the two primary histological groups of breast cancer, with approximately 70–80% of all cases falling into one of these categories [15,16]. Deep-learning (DL) methods are capable of autonomously extracting features, retrieving information from data, and learning sophisticated abstract interpretations of the data. DL techniques are powerful. They can resolve typical feature-extraction issues and have found use in a selection of sectors, including computer vision and biomedicine.

**Figure 2:**
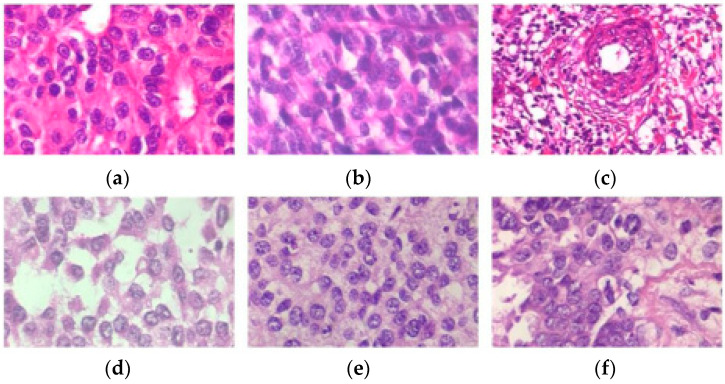
Samples (**a**–**e**) are ductal carcinomas (DCs), while sample (**f**) is a phyllodes tumor carcinoma (PTC) from a woman with breast cancer. Each image is a 400× magnification from the BreakHis archive.

**Figure 3:**
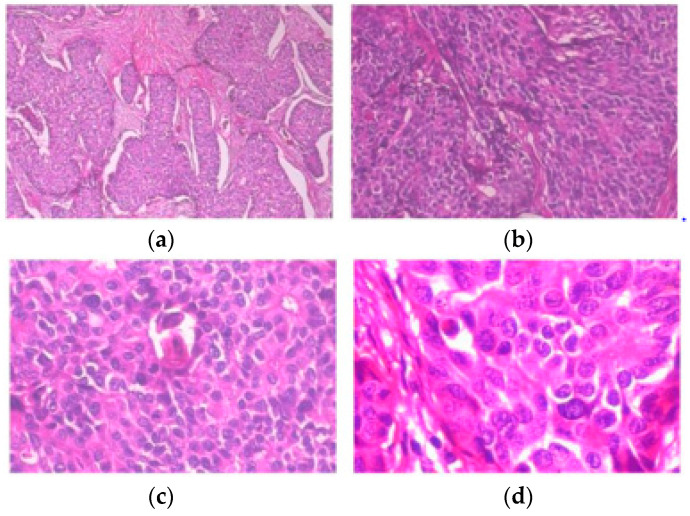
Slides of breast ductal carcinoma from the same patient at (**a**) 40×, (**b**) 100×, (**c**) 200×, and (**d**) 400×. BreakHis has provided the images [20].

Centered on deep convolutional neural networks, a new BC histopathological image category blind inpainting convolutional neural network (BiCNN) model has been developed. It was developed to cope with the two-class categorization of BC on the diagnostic image. The BiCNN model uses previous knowledge of the BC class and subclass labels to constrain the distance between the characteristics of distinct BC pathology images [17]. A data-augmented technique is provided to suit the acceptance of whole-slide image identification [18]. The transfer-fine-tuning training approach is employed as an appropriate training approach [19] to increase the accuracy of BC histological image categorization. Figure 2 and Figure 3 demonstrate some of the finer characteristics of the pathological images of BC. Samples (a) through (e) in Figure 2 are all ductal carcinomas (DCs). The phyllodes tumor is sample (f). The colors and forms of the cells in samples (a)–(e) all belong to DCs, even though they are all DC samples. Samples (e) and (f) have a striking resemblance in terms of color and cell shape; however, they are classified as distinct classes. Figure 3 depicts abnormal images at various magnification levels. There is a substantial variance in the visual features across the various magnifications, even though they are all from the same subject [20].

## 2. Literature Review

The National Institute of Oncology in Rabat, Morocco, received 116 surgical breast specimens with invasive cancer of an unknown nature, resulting in 328 digital slides. These photos were properly classified into one of three types: normal tissue–benign lesions, in situ cancer, or aggressive carcinoma. It was shown that, despite the small size of the dataset, the classification model developed in this research was able to accurately predict the likelihood of a BC diagnosis [21]. To compare the performance of chronic myelogenous leukemia (CML)- and DL-based techniques, the author also provided a visual analysis of the histological results to categorize breast cancer. CML-based approaches utilize three feature extractors to extract hand-crafted features and combine them to build an image representation for five traditional classifiers. The DL-based techniques utilized the well-known VGG-19 DL design, which was fine-tuned using histopathological images. The data showed that the DL methods outperformed the CML methods, with an accuracy range of 94.05 to 98.13% for the binary classification and 76.77 to 88.95% for the eighth-class classification [22]. The DCNN-based heterogeneous ensemble method for mitotic nuclei identification was used for breast histopathology images using the DHE-Mit-Classifier. Histopathological biopsy samples were examined for the presence of mitotic patches, and the DHE-Mit-Classifier was used to sort them into mitotic and nonmitotic nuclei. A heterogeneous ensemble was constructed using five independent DCNNs. the mitotic nuclei’s structural, textural, and morphological characteristics remain captured by these DCNNs, which included a variety of architectural styles. The recommended ensemble had an F-score of 0.77, a recall of 0.71, a precision of 0.83, and an area under the curve (AUC) accuracy–recall of 0.83, which surpassed the test set of 0.80. The F-score and accuracy indicated that this ensemble might be utilized to build a pathologist’s helper [23]. The BC patients benefited from the enhanced and multiclass whole-slide imaging (WSI) segmentation uses of the CNN. These components organize information collected from CNNs into pathologists’ predictions. Pathologists need instruments that can speed up the time to perform histological analyses, provide a second opinion, or even point out areas of concern during routine screening. This yielded a sensitivity of 90.77%, a precision of 91.27%, an F1 score of 84.17%, and a specificity of 94.03%. The area subdivision module acquired a sensitivity of 71.83%, an IOU of 88.23%, an intersection over union (IOU) of 93.43%, a precision of 96.10%, an F1 score of 82.94%, a specificity of 96.19%, and an AUC of 0.88 for the improved WSI segmentation [24]. A hybrid model based on DCNNs and pulse-coupled neural networks (PCNNs) was developed. Transfer learning (TL) was used in this study due to the necessity for huge datasets to train and tune the CNNs, which were not accessible for medical images. TL can be an efficient method when dealing with tiny datasets. The document’s application was assessed using three public standard datasets, DDMS, INbreast, and BCDR, for the instruction and analysis, and MIAS for testing alone. The findings demonstrated the benefit of combining the PCNN with the CNN over other approaches for the same public datasets. The hybrid model accurately predicted DDMS (98.72%), BCDR (96.94%), and breast cancer (97.5%). The proposed hybrid model was tested on a previously unreported MIAS dataset and showed an accuracy of 98.7%. In the Results section, further assessment measures can be found [25]. There are a variety of digital pathology image-evaluation techniques for breast cancer, including rule-based and machine-learning approaches [26]. Lately, DL-based processes have been proven to outpace traditional machine-learning techniques in several image-evaluation tasks, computerizing the whole-processing process [27]. Convolution neural networks (CNNs) have been utilized effectively in the medical imaging field to detect diabetic retinopathy, forecast bone disease and age, and other issues. Earlier DL-based functions in histological microscopic image processing have shown their ability to be useful in the diagnosis of breast cancer. The detection of BC has become more dependent on machine learning over the last several decades. The machine-learning method includes a variety of probabilistic, statistical, and optimization techniques for deriving a classification model from a dataset [28].

## 3. Research Methodology

This section explains the suggested methodology of the research work. In this methodology, the procedure is categorized into four categories: visual synonyms, image segmentation, similarity, and model training. DML and label propagation are two techniques that are employed in this approach for explainable BC histopathological image diagnosis. This process includes a draught for visual synonyms with a fuzzy set of criteria, as well as the generation of real synonyms and the expansion of the keyword list. An image dataset is used, which was generated, that contains the following elements: image preprocessing, revision and normalization of the input image, image segmentation, and the segment difference score using label propagation. After completing the image segmentation and visual synonym processes, it generates the similarity between them; finally, it performs model training with the aid of the DML approach.

The techniques used in the proposed methodology are discussed below.

### 3.1. Training Based on DML

The actual process used to autonomously train the proposed model is described as the subsequent multiclass cross-entropy loss:

(1) $$L_{C}=-(y_{C}\cdot log\left(\emptyset_{C}\right)\left(1-y_{C}\right)\cdot log\left(1-\emptyset_{C}\right))$$

where $\emptyset_{C}$ is the possibility that a bag is expected as a positive classification and $y_{C}\in$ 0, 1 suggests that a histopathological image is expected to be malignant or benign.

**Figure 4:**
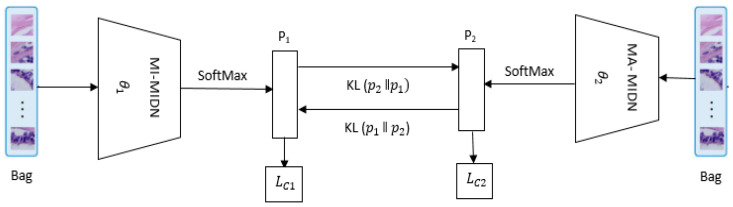
The DML schema.

**Figure 5:**
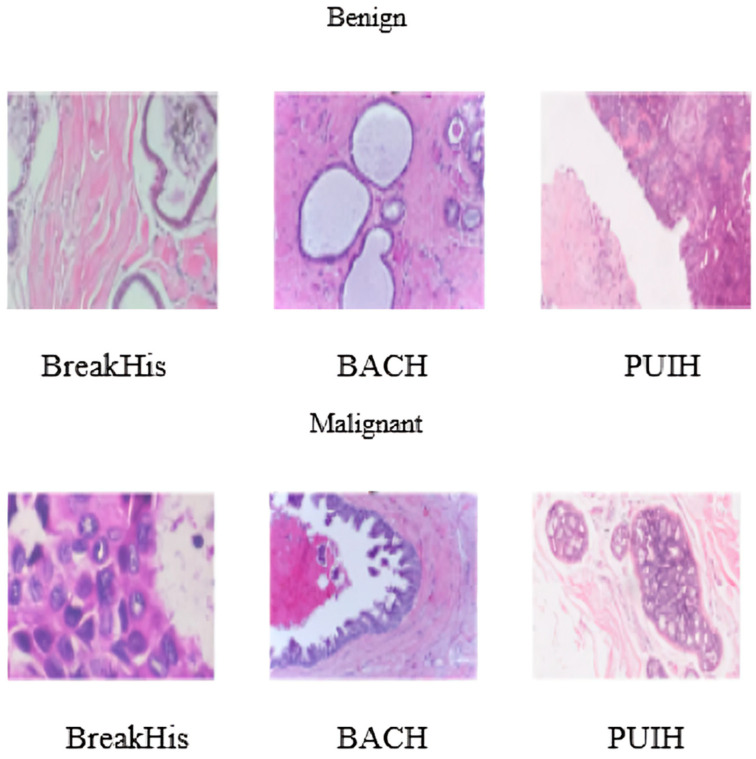
Images from three publicly available datasets illustrating histopathology.

The analysis model is accomplished separately in typical circumstances, which does not allow for the diagnostic model’s full potential to be tapped. The goal is to train two models in a cohort using the DML schema as shown in Figure 4. [29]. As illustrated in Figure 5, $\theta_{1}$ and $\theta_{2}$ are two indistinguishable entities (networks) of the model. Two same bags are input into the DML structure at a similar moment; $P_{1},P_{2}\in R^{2\times 1}$ are the outputs of every individual network.

Let $P_{1}^{1}$ and $P_{1}^{1}$ signify the possibility that $\theta_{1}$ forecasts that a bag goes to the positive and negative classes, separately. The *KL* distance from $P_{1}$ to $P_{2}$ is calculated as

(2) $$D_{KL}=P_{2}^{0}log\frac{P_{2}^{0}}{P_{1}^{0}}+P_{2}^{1}log\frac{P_{2}^{1}}{P_{1}^{1}}$$

The total loss functions $L_{\theta_{1}}$ and $L_{\theta_{2}}$ for the *θ*_1_ and *θ*_2_ networks are thus achieved, correspondingly, as follows:

(3) $$L_{\theta_{1}}=L_{C_{1}}+D_{KL}(P_{2}ǁP_{1})$$

(4) $$L_{\theta_{2}}=L_{C_{2}}+D_{KL}(P_{1}ǁP_{2})$$

The loss *LC* and the *KL* mimicry loss teach every network to forecast the correct description of the input bag and to match the possible value of its peer network. They can convert the initial DML schema from supervised to inadequately supervised learning in this way. Furthermore, the DML architecture allows for bidirectional information transfer via collective training in a cohort, as well as the ability to tap into the model’s capacity for the accurate categorization of the histopathology images [30].

BreakHis, BACH, and PUIH are all publicly accessible BC histopathology image datasets used to validate the proposed DML model. There are 7909 histopathological images in the BreakHis dataset, each with three channels and four magnifications. PUIH has 4020 three-channel images, while BACH contains 400. The magnification of the images in these two datasets is not specified. BACH and PUIH include 2048 × 1536 pixel images, while BreakHis has images that are 700 × 460 pixels in size. An in-depth look at the three datasets is provided in Table 1. Figure 5 shows a selection of these photos.

### 3.2. Label Propagation for Image Segmentation

Label propagation is a semisupervised machine-learning method that adds labels to data points that were previously unlabeled. Image segmentation is an essential component of various image-processing systems. Few computerized image-analysis approaches can be used autonomously with good results in most circumstances [31].

The term interactive segmentation comes from the fact that semiautomated segmentation algorithms enable users to engage in the segmentation process and provide some direction for the description of the required material to be retrieved [32]. An interactive segmentation algorithm that works in practice must have four qualities: quick calculation, quick editing, the capacity to create arbitrary segmentation given enough interactions, and understandable segmentation. Active-contour- or level-set-based approaches, as well as graph-cut-based methods, have been presented in the recent several decades for image segmentation.

Although these algorithms have been successful in many circumstances, there are still a few issues with their use. It is difficult to execute the level-set-based or active-contour solutions; subsequently, the user must input the many free factors. The graph-cut-based systems only replace the tiniest cut that separates the seeds (i.e., the labeled pixels), and they typically provide the tiny reductions that simply divide the seeds from the remaining pixels when the number of seeds is extremely small.

### 3.3. Proposed Methodology

**Figure 6:**
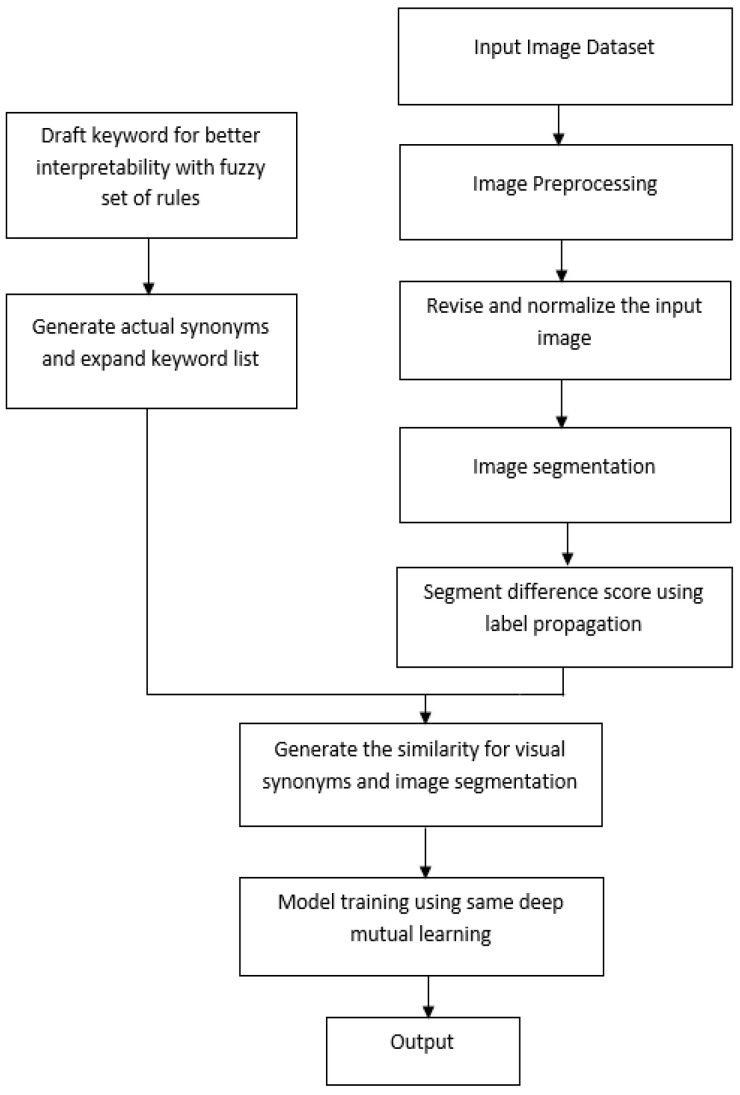
Block diagram of the proposed methodology.

This section provides the in-depth detail of the proposed methodology. The proposed methodology block diagram is shown in Figure 6 below:

Step 1: Input Image Dataset

This is the collection of images with which the analysis will be performed.

Step 2: Image Preprocessing

The initial step involves revising and normalizing the input images. This could include tasks like resizing, cropping, or adjusting the color levels to prepare the images for further analysis.

Step 3: Image Segmentation

Image segmentation involves dividing an image into different segments to identify and analyze different regions. This can be useful for tasks like object recognition or scene understanding.

Step 4: Segment Difference Score using Label Propagation

This step suggests assigning scores to the segmented regions, possibly using a label-propagation technique. Label propagation is a semisupervised learning method that can be used to propagate labels from a small set of labeled data to unlabeled data.

Step 5: Draft Keyword for Better Interpretability with a Fuzzy Set of Rules

This step involves generating keywords that help in interpreting the results. Fuzzy set theory might be employed here to handle uncertainty in the data.

Step 6: Generate Actual Synonyms and Expand the Keyword List

This step implies creating synonyms for the drafted keywords and expanding the keyword list to capture a broader range of concepts related to the analysis.

Step 7: Generate Similarity for Visual Synonyms and Image Segmentation

This involves assessing the similarity between visual synonyms (possibly the segmented regions) and the results of the image segmentation.

Step 8: Model Training Using the Same Deep Mutual Learning

Deep mutual learning usually refers to training models collaboratively. In this context, it suggests training a model using the information gained from the image segmentation and the fuzzy set of rules.

Step 9: Output

Based on the training, the output of the model is generated in this step, which is associated with the state-of-the-art technique based on various parameters (AUC, precision, recall).

These steps are summarized as Algorithm 1.
**Algorithm 1 Deep Mutual Learning for Breast Cancer Histopathology Image Diagnosis****Require**: Image datasets (BreakHis, BACH, PUIH)**Ensure**: Trained model for breast cancer histopathology image diagnosis1: Initialize two identical neural network models *θ*_1_; and *θ*_2_2: **for** each batch of input images **do**3:   Preprocess images (revision, normalization)4:   Perform image segmentation5:   Calculate segment difference score using label propagation6:   Compute multi-class cross-entropy loss *L_C_* for each model7:   *L_C_* = −(*y_C_* · *log*(Φ_*C*_) + (1 − *y_C_*) · *log*(1 − Φ*_C_*))8:   Calculate KL divergence *D_kL_*, between the two models9:   *D_KL_* = *P*_2_^0^ · *log*(*P*_2_^0^/*P*_1_^0^) + P_2_^1^ · *log*(*P*_2_^1^/*P*_1_^1^) 10:    Update total loss functions for each model11:    *L*_*θ*1_ = *L*_*C*1_ + *D_KL_*(*P*_2_ || *P*_1_)12:    *L*_*θ*2_ = *L*_*C*2_ + *D_KL_*(*P*_1_ || *P*_2_)13:    Train both models using the computed losses14: **end for**15: Repeat steps 2–11 until convergence16: Evaluate the trained model on test datasets

## 4. Results

In this part of the study, the implementation that was carried out utilizing the technique that was suggested is presented. MATLAB 2020 (Mathworks India Private Limited, Bangalore, India) was used as a functional tool.

The BreakHis, BACH, and PUIH BC histopathology image datasets were used to test the suggested DML. A receiver operating characteristic (ROC) curve was used to assess the suggested model’s accuracy objectively and fully, among other evaluation criteria, i.e., the AUC.

**Figure 7:**
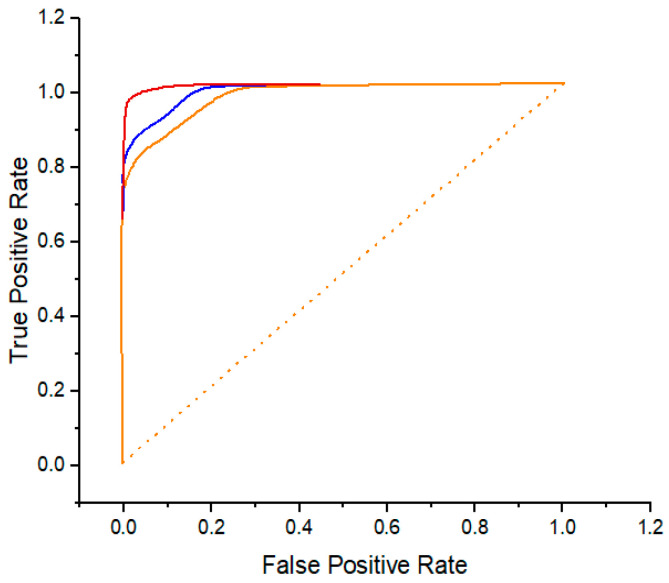
ROC curve for the classification results using the BreakHis-200× dataset.

**Figure 8:**
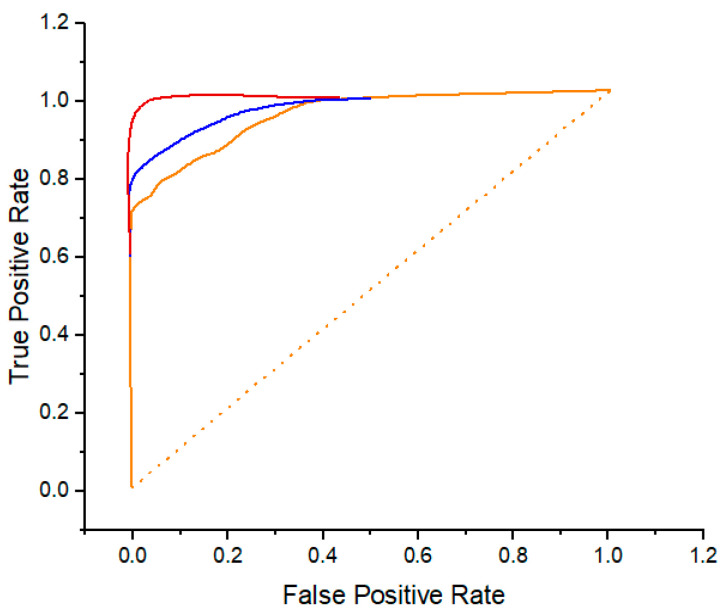
ROC curve for the classification results using the BACH dataset.

**Figure 9:**
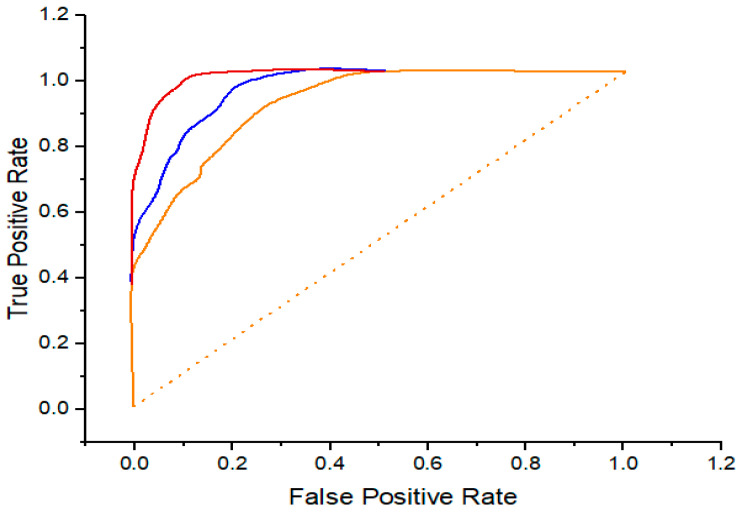
ROC curve for the classification results using the PUIH dataset.

Figure 7, Figure 8 and Figure 9 demonstrate the parallel ROC curve and AUC value on every dataset. The results that are shown in Figure 8 illustrate the ROC curves and the AUC values for the BreakHis-200× dataset. The MA-MIDN-Ind model and the MA-MIDN-DML model lag behind the DML model. Considering all of them, it seems that DML performs a role that is energetic. In addition to enhancing the accuracy of the final organization, it could additionally validate the potential of the model to simplify complex situations by using the BreakHis dataset.

The ROC curve and the area under the curve (AUC) value for the BACH dataset are shown in Figure 8, which describes the outcome. It is of the greatest significance that the DML on the BACH dataset be accurate when it comes to the MA-MIDN model (both the MA-MIDN-DML model and the MA-MIDN-Ind model simultaneously). These data can be made use of in order to demonstrate and enhance the generalization capabilities of the model.

Figure 9 displays the ROC curve and AUC value for the PUH dataset. The AUC of the DML using the PUIH dataset is critical in the MA-MIDN model’s performance (the MA-MIDN-DML model and MA-MIDN-Ind model). It has the potential to demonstrate the correctness of the model while also increasing the generalization capacity of the model.

**Figure 10:**
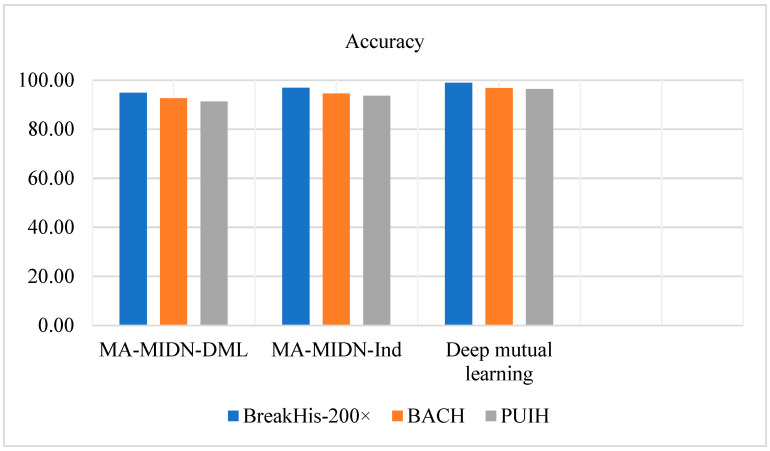
Comparison of the DML with the independent and DML-based training systems for accuracy.

Table 2 illustrates the comparison of the current methodologies with the proposed methodology. Table 2 and Figure 10 show that the proposed DML model beats the MA-MIDN-DML model and the MA-MIDN-Ind model by a large margin on the BreakHis, BACH, and PUIH datasets.

**Figure 11:**
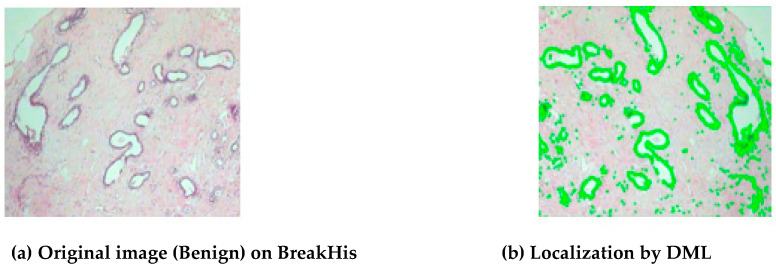
MA-MIDN model’s localization output on three publicly available datasets.

**Figure 12:**
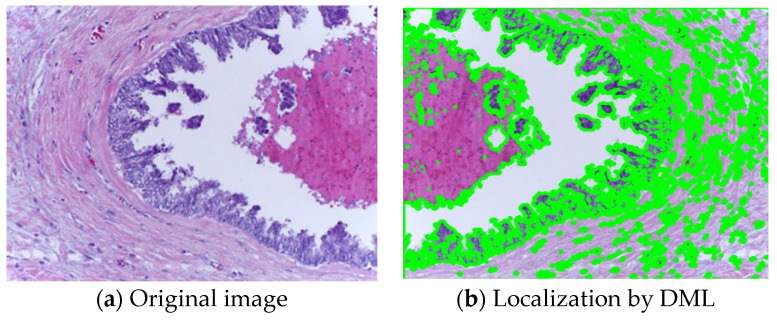
Different attention processes’ localization outcomes on the BACH dataset.

Figure 11 displays the findings for all datasets in terms of localization. The objective and complete evaluation of the obtained localization findings is based on both benign and malignant images with varying morphologies. Figure 11a shows the original image on BreakHis dataset and Figure 11b shows the localization outcome by DML on BreaKHis dataset. Figure 12 shows different attention processes’ localization outcomes on the BACH dataset. Figure 12a shows the original image of BACH dataset and Figure 12b shows the Localization outcome by DML on BACH dataset.

A variety of current methodologies are examined on each dataset to see how well the suggested model performs. On the BreakHis dataset, we first evaluated our model compared to the following baseline styles: Res Hist-Aug, FCN + Bi LSTM, MI-SVM, Deep MIL, and the MA-MIDN model. Table 3 shows the results of the accuracy comparisons.

Table 4 shows the differences of UC, precision, recall and F1 on Breakhis dataset. As compared to BreakHis, BACH, and PUIH (the most recent dataset to be published in 2020), the images have a greater resolution. So, the DML model has a major hurdle in classifying the two datasets. The Patch + Vote, B + FA + GuSA, Hybrid-DNN, and MA-MIDN are the baseline models against which we compared our model on the BACH and PUIH datasets. The comparisons of the performances are shown in Table 5.

**Figure 13:**
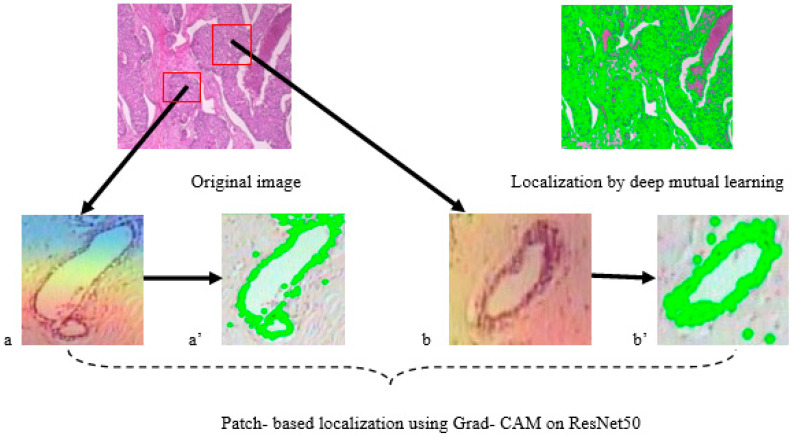
Evaluation of the DML and Grad-CAM for localization.

The Grad-CAM approach was used in conjunction with the ResNet50 model, which was trained on the BACH dataset. Figure 13a,b shows the breast glands from the original image of BACH dataset. Figure 13a’,b’ shows the GRAD-CAM patch based localization findings with ResNet50 model.

**Figure 14:**
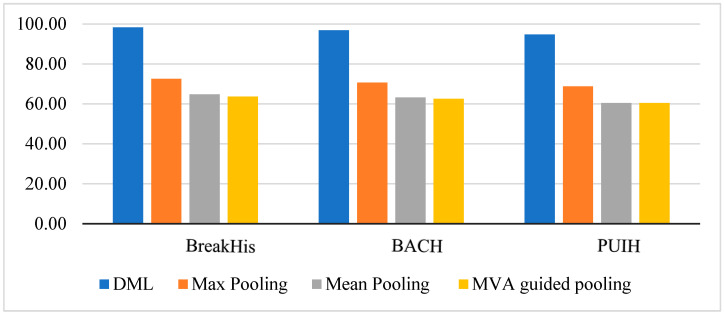
Performance associations between the suggested methods and the other pooling methods.

The DML was compared to the other popular pooling approaches. It directly performs max and means pooling on instance-level features to achieve the test results. Figure 14 displays the results of the analysis.

The appropriate experimental outcomes are shown in Table 6, employing the MI-Net and running tests on three datasets; “No Attention” yields its findings [36].

The DML model’s localization results consume most of the testing time, as shown in Table 7 and Table 8. This dataset has a lower image size than the previous two; therefore, the DML model runs quicker on it. For the BACH and PUIH datasets, the average classification time of the DML model was 0.09 s, while the average classification time for simultaneous localization was 1.55 s. These numbers are satisfactory to a certain degree, but they could be improved upon further.

A unique multiview attention-guided multiple-instance detection network (MA-MIDN) is presented to address this issue. Multiple-instance learning (MIL) can be used to solve the classic image-categorization issue. It first separates each histopathological image into instances and then builds a matching bag to obtain the maximum use of the high-resolution data provided by the MIL. A novel multiple-view attention (MVA) technique is presented to train the awareness of the occurrences in the image to identify the wound locations in this image. An MVA-guided MIL sharing technique is intended to aggregate instance-level characteristics to acquire the bag-level characteristics for the last organization. The suggested MA-MIDN standard operates image classification as well as lesion localization at the same time. The MA-MIDN model is specifically trained using DML. DML is now a poorly supervised learning issue. Three community BC histopathology image datasets were used to test the categorization and localizations findings. The investigational findings indicate that the MA-MIDN model outperforms the most recent criteria in conditions of diagnostic precision, AUC, recall, precision, and the F1-score. Specifically, it delivers improved localization outcomes without sacrificing categorization performance, indicating its greater usefulness [29].

## 5. Conclusions and Future Scope

Advances in DL techniques have proven a substantial improvement in the diagnosis of BC histopathology images. Even with the use of high-resolution histopathology images, training and interpretable diagnostic models remain a difficult endeavor. The DML approach is being considered to ease this difficulty. Compared to the prior approach on three datasets (Break His, BACH, and PUIH), the suggested technique outperforms the previous technique. The accuracy of the suggested approach shows that the DML model, in the terms of Break His-200×, BACH, and PUIH datasets (98.97%, 96.78%, and 96.34%), outperform the highest value of the current techniques. The proposed approach provides for quicker data transfer, since it reduces the propagation delay. As a result, the suggested approach outperformed the current technique when compared to it.

The proposed model could be further expanded in the future to provide better functionality in terms of protecting the confidentiality of users and providing the quality of data collected by medical institutions. Its goal is to enhance the implementation of the diagnostic model to make it useful in clinical practice.

## Figures and Tables

**Table 1:** The following is a summary of three publicly available datasets.

Dataset	Image Size	Magnification Factor	Benign	Malignant	Total
BreakHis	700 × 460	40×	625	1370	1995
		100×	644	1437	2081
		200×	623	1390	2013
		400×	588	1232	1820
BACH	2048 × 1536	_	200	200	400
PUIH	2048 × 1536	_	1529	2491	4020

**Table 2:** Comparison of the individual and DML-based training systems by accuracy.

Training Methods	BreakHis-200×	BACH	PUIH
MA-MIDN-DML [33]	94.90	92.67	91.33
MA-MIDN-Ind [33]	96.87	94.54	93.65
Proposed DML	98.97	96.78	96.34

**Table 3:** Precision differences on the BreakHis dataset; unit: %.

Methods	40×	100×	200×	400×	Mean
Res Hist-Aug [34]	90.42	90.87	93.88	89.54	90.89
FCN + Bi-LSTM [35]	95.78	94.51	97.23	94.30	95.89
MI-SVM [36]	86.44	82.90	81.75	82.78	83.45
Deep MIL [37]	91.92	89.66	91.78	85.99	89.84
MA-MIDN [33]	96.56	96.99	97.88	95.66	89.83
DML	97.87	98.56	98.34	96.54	93.37

**Table 4:** Differences of AUC, Precision, Recall, and F1 on the BreakHis dataset; unit: %.

Methods	Magnification Factor	AUC	Precision	Recall
Res Hist-Aug [34]	40×	94.67	93.77	87.21
	100×	93.22	90.44	89.44
	200×	94.89	94.26	92.69
	400×	95.34	91.45	86.89
MA-MIDN [33]	40×	95.56	95.78	88.87
	100×	94.43	92.19	90.63
	200×	95.67	94.89	94.78
	400×	96.33	93.67	91.56
DML	40×	97.89	97.56	92.56
	100×	98.34	95.38	95.45
	200×	97.89	98.65	98.31
	400×	99.44	99.71	96.44

**Table 5:** Performing similarities on the BACH and PUIH datasets; unit: %.

Datasets	Methods	Accuracy	AUC	Precision	Recall
BACH	Patch Vote [38]	86.22	92.29	86.98	81.97
	B + FA + GuSA [39]	91.35	96.67	95.78	86.67
	MA-MIDN [33]	94.67	97.98	96.45	95.26
	DML	97.46	98.67	97.89	96.36
PUIH	Hybrid-DNN [40]	92.25	-	-	-
	MA-MIDN [33]	93.76	97.26	95.07	95.19
	DML	95.56	98.67	96.83	97.17

**Table 6:** Precision evaluations among various attention mechanisms; unit: %.

Attention Mechanisms	BreakHis-200×	BACH	PUIH
No Attention (MI-Net) [41]	76.47	71.67	70.43
Attention over instances (AOIs)	74.78	69.34	67.73
Attention over classes (AOCs)	88.28	83.88	81.71

**Table 7:** The average amount of time it takes to run a single classification test on each batch.

Datasets	Average Test Time of Each Image in Each Batch					
	1	2	3	4	5	Mean
**BreakHis-200×**	0.04	0.03	0.03	0.03	0.04	0.03
**BACH**	0.09	0.09	0.11	0.09	0.10	0.09
**PUIH**	0.10	0.11	0.10	0.10	0.09	0.10

**Table 8:** Each batch’s average testing time for classification and localization.

Datasets	Average Test Time Each Image in Each Batch					
	1	2	3	4	5	Mean
**BreakHis-200×**	0.82	0.67	0.70	0.67	0.67	0.71
**BACH**	1.44	1.66	1.66	1.36	1.61	1.55
**PUIH**	1.43	1.56	1.46	1.62	1.55	1.52

## Data Availability

The BreakHis dataset is available at: https://web.inf.ufpr.br/vri/databases/breast-cancer-histopathological-database-breakhis/ (accessed on 5 July 2022). The BACH dataset is available at: https://zenodo.org/records/3632035 (accessed on 5 July 2022).
